# Forest stand factors determine the rainfall pattern of crown allocation of *Picea schrenkiana* in the northern slope of Mount Bogda, Tianshan Range, China

**DOI:** 10.3389/fpls.2022.1113354

**Published:** 2023-01-31

**Authors:** Shanchao Zhao, Xin-Jun Zheng, Lihe Yin, Yugang Wang

**Affiliations:** ^1^ Natural Forest Protection Center of Xinjiang Uygur Autonomous Region, Urumqi, China; ^2^ State Key Laboratory of Desert and Oasis Ecology, Xinjiang Institute of Ecology and Geography, Chinese Academy of Sciences, Urumqi, Xinjiang, China; ^3^ Fukang Station of Desert Ecology, Chinese Academy of Sciences, Fukang, Xinjiang, China; ^4^ Xi’an Center of Geological Survey, China Geological Survey, Ministry of Natural Resources, Xi’an, Shaanxi, China; ^5^ University of Chinese Academy of Sciences, Beijing, China

**Keywords:** biomass carbon density, interception, *Picea schrenkiana*, stemflow, throughfall, crown rainfall allocation

## Abstract

The middle elevation forest of the Tianshan Mountains, dominated by the conifer tree *Picea schrenkiana*, is an important part of the mountain ecosystem of arid Northwestern China, which plays a pivotal role in carbon sequestration and water conservation. As the first interface of water transfer in a forest ecosystem, tree crown allocates precipitation regulating soil water supply and sustaining vegetation growth below the crown. In this study, four 20-m × 20-m sampling quadrats were randomly installed at each of three elevation sites (2,200 m, 1,800 m, and 1,450 m) on the northern slope of Mount Bogda, the main peak of the Eastern Tianshan Range. The effects of forest stand factors and incoming rainfall on forest crown allocation of precipitation were investigated, and the trade-off between water and carbon was also discussed. The results revealed that (1) the interception, throughfall, and stemflow ratio had values of 44.3%–50.0%, 49.6%–55.4%, and<0.5%, respectively; (2) there was a complementary relationship between stemflow ability and threshold rainfall when stemflow emerged, and the crown interception rainfall had a saturation value; and (3) the allocation of crown-intercepted rainfall was controlled by trunk diameter at breast height, crown height-to-width ratio, and leaf area index, which was why differences arose in the allocation of crown precipitation at differing elevations. With greater arbor biological carbon density, the crown interception ratio initially increased rapidly but then remained stable, indicating that once a natural forest stand is mature, its biomass carbon sequestration would not change further allocation of crown precipitation.

## 1 Introduction

*Picea schrenkiana*, the most important zonal montane forest species of Tianshan Mountains, Central Eurasia, is widely distributed at elevations of 1,400–2,800 m on the windward slope (Li et al., 2018a; Adilai et al., 2021; Li et al., 2021). This spruce forest accounts for more than 95% of the total forest resources in Tianshan Mountains, 59% of its mountainous forest area, 44.9% of its total natural forest area, and 60.8% of the timber volume in Xinjiang of China (Liu et al., 2011; Zhang et al., 2016a; Jiao et al., 2019). Net primary productivity of this Tianshan Mountains–spruce forest was estimated to be 0.9 t hm^−2^ a^−1^ (Ni, 2004) whereas its vegetation carbon storage was estimated at 53.14 Tg C (Wang et al., 2001b; Xu et al., 2016; Li et al., 2018b). Thus, given the still rapidly rising carbon emissions of Xinjiang (Yin et al., 2021), the Tianshan Mountains–spruce forest is of great significance for Xinjiang’s ability to achieve the twin goals of “peak carbon” and “carbon neutrality”.

Meanwhile, the alpine spruce forest is also the main component of the mountain ecosystem in the arid and semi-arid areas of Northwest China (Luo et al., 2020). It has multiple ecological functions, such as wind prevention and dust capture, soil and water conservation, water source conservation, water quality purification, land erosion mitigation, climate regulation, and biodiversity protection, which is why it is considered a crucial ecological security pillar in Arid Northwestern China (Wang et al., 2017; Lan et al., 2020). For all of those reasons, in 2021, the central government of China promulgated the “Key Construction Program of Ecological Protection and Restoration in Northern Shelterbelt (2021–2035)”, where protecting the forests and grasslands in Tianshan and Altai mountains was deemed an imperative task. Its paramount goal is promoting the management and protection level of alpine spruce forests, which entails developing water source conservation forests and strengthening forest tending, thereby improving the overall quality of forest resources. Therefore, we should pay attention not only to the direct benefits that forests can provide in terms of wood, biochar, and the other forest products, but also to the indirect benefits of ecosystem services with respect to climate regulation, water source conservation, and flood disaster retention and reduction, all of which depend on the dynamic relationship between forest and water (Bosch and Hewlett, 1982). To sum up, it is necessary to conduct in-depth research on forest eco-hydrological processes and to evaluate their environmental benefits, at whose core is the relationship between vegetation cover and runoff, to elucidate the mechanisms underpinning allocation of crown-intercepted precipitation, water retention by litter, soil water infiltration and storage, terrestrial evapotranspiration, and so on, among forest strata such as vegetation crown, ground covering layer of plants, and the soil (Wang et al., 2001a; Xian et al., 2014; Lu et al., 2015; Liu et al., 2022).

As such, the eco-hydrological processes of mountainous forests, including crown interception, stemflow, surface runoff, and soil infiltration, all feature prominently in water resource conservation (Ran et al., 2012). The absence and imbalance of functions and processes are the major causes of soil erosion and land degradation in mountainous areas (Wang et al., 2019; Sun et al., 2021). The forest crown allocation of precipitation is a very important eco-hydrological process that also affects the forest water balance and geochemical cycling; hence, it is ecologically significant for multiple reasons (Llorens and Domingo, 2007; Peng et al., 2014; Van Stan and Pypker, 2015; Liu et al., 2016). *Via* allocation by the forest crown, rainfall is partitioned into three components: throughfall, stemflow, and crown interception. Throughfall and stemflow can reach the ground surface and enter soil, replenishing soil moisture, which can be taken up by plant roots, and thus considered effective rainfall. Crown interception, however, is long believed to return to the atmosphere as evaporation, meaning it is ineffective rainfall (Park and Cameron, 2008; Staelens et al., 2008). Yet, crown interception can be effective precipitation due to the since confirmed existence of water absorption on the leaf surface (Breshears et al., 2008; Zheng et al., 2011). The process of forest crown allocation of precipitation is affected by the characteristics of both rainfall and the intercepting tree crown, and their combined effects with the other meteorological factors, such as temperature, humidity, wind speed, and net radiation, as well as their interactions. Rainfall characteristics include the amount, intensity, and duration of water, and their spatiotemporal distributions, whereas crown characteristics include crown density, crown structure, crown dryness, age, trunk diameter at breast height (DBH), tree height, leaf shape, branching angle, leaf area index (LAI), and the gap fraction (Levia and Frost, 2003; Llorens and Domingo, 2007; Park and Cameron, 2008; Staelens et al., 2008; Germer et al., 2010; Siegert and Levia, 2014; Sun et al., 2014; Zhang et al., 2015b; Zimmermann et al., 2015; Zhang et al., 2016b; Zhang et al., 2019).

The crown constitutes the first interface of water transfer and movement in a forest ecosystem, and the ensuing allocation of rainfall regulates the moisture distribution pattern of forest cover and soil layer, which has an important impact on biodiversity of forests and their runoff yield (Zhao et al., 2002; Dang et al., 2005). This regulation of water flow by the crown is mainly determined by the amount of rainfall and stand factors including LAI, tree species identity, tree height, and the width and density of the crown (Siegert and Levia, 2014; Van Stan and Pypker, 2015). Therefore, given that the amount of the precipitation pulse and forest stand factors are likely common decisive factors governing the forest crown allocation of precipitation, the following hypothesis was put forward: Differences in crown rainfall allocation among different studied forest plots are determined by stand factors, with stand development leading to an altered coupling and trade-off relationships between biological carbon sequestration and water resource conservation and other ecosystem services in dryland mountainous forest key to water conservation.

## 2 Study area and methodology

### 2.1 Study area

This study area is located in the Fukang state-owned forest region, where the north slope of Mt. Bogda, the main peak of the eastern Tianshan Mountains, is found. It is a typical complete continental mountainous landscape region of Inner Eurasia, which integrates glaciers, snowpack, forest, grassland, and alpine lakes (Zhou et al., 2009). The total annual sunshine duration is 2,600 h and the average annual temperature is below 1.9°C; the winter is long and cold with –12.4°C being the average temperature in the coldest month, while the summer is short and warm with 15.9°C being the average temperature of the hottest month (Wang et al., 2009; Wang et al., 2015). The weak wet and cold water vapor from the North Atlantic Ocean and the Arctic Ocean brought by the westerly circulation in the Northern Hemisphere is blocked and uplifted by the tall mountains of the Tianshan Mountains, forming topographic rain on the northern slope of the windward face (Hu et al., 2014), enabling the average annual precipitation in this region to reach 520.6 mm (Ma et al., 2010). The soil here consists mainly of taupe alpine forest gray-brown soil, with a thin layer of forest residue covering the ground surface, followed by humus layer and leaching layer, and gradually transitions to a sedimentary clay layer that contains white pseudomycelial calcium carbonate deposition nuclei. The vegetation consists mainly of pure spruce stands of *P. schrenkiana* in temperate mountain coniferous forest. However, one can find scattered trees such as *Populus talassica*, *Sorbus tianschanica*, and *Betula tianschanica* in the forest margins and gaps. In the sparse forest understory are the shrubs *Cotoneaster melanocarpus*, *Berberis heteropoda*, *Rosa spinosissima*, *Spiraea hypericifolia*, *Sabina pseudosabina*, and *Lonicera hispida*, and herbaceous plants such as *Alchemilla tianshanica*, *Geranium divaricatum*, and *Aegopodium podagraria*.

### 2.2 Study quadrat, vegetation survey, and observation

#### 2.2.1 Study quadrat, vegetation survey, leaf area index, and arbor biomass carbon density

Three sites (denoted A–C) were studied at 2,200-m, 1,800-m, and 1,450-m elevations, respectively located at Natazi (43°56′9″N–43°56′27″N, 88°13′12″E–88°13′22″E), the upper part (43°55′60″N–43°56′01″N, 88°9′2″E–88°9′17″E), and the entrance (43°56′7″N–43°56′14″N, 88°6′30″E–88°7′17″E) of Baiyanggou of the Fukang state-owned forest region (**Table 1**). At each site, four sapling quadrats (20 m × 20 m) were randomly positioned and set up. The DBH (cm), crown radius (CR, m), and tree height (*H*, m) of all trees and the population density of tree individuals (PD, hm^−2^) in every quadrat were measured and recorded. The crown area (CA, m^2^) and crown height-to-width ratio (HWR) of each tree were as follows:

**Table 1:** Mean ( ± SD) of diameter at breast height (DBH, cm), crown height-to-width ratio (HWR, m/m), leaf area index (LAI) and population density (PD, hm^−2^), and arbor biomass carbon density (CD, t C hm^−2^) of *Picea schrenkiana* in the sites of 2,200-m, 1,800-m, and 1,450-m elevations at the northern slope of Mount Bogda, Tianshan Range, Xinjiang, China.

Study site	Elevation (m)	*N*	DBH (cm)	HWR (m/m)	LAI	PD (hm^−2^)	CD (t C hm^−2^)
A	2,200	4	29.4 ± 2.1	8.0 ± 1.2	1.09 ± 0.61	681 ± 244	48.5 ± 27.9
B	1,800	4	26.4 ± 5.7	6.9 ± 2.2	1.04 ± 0.84	813 ± 136	46.2 ± 41.8
C	1,450	4	37.9 ± 5.1	8.6 ± 0.7	0.62 ± 0.04	369 ± 55	32.4 ± 2.4


(1) $$\text{CA}=\text{π}\cdot {\text{CR}}^{2}$$

(2) HWR=H/(2×CR)

The quadrat-level average LAI was measured at the height of 1.5 m in later summer using the plant canopy analyzer of LAI-2000 (LI-COR, Lincoln, Nebraska, USA). The trunk, branch, root, and leaf biomass (BM_t_, BM_b_, BM_r_, and BM_l_, kg) of each individual tree were calculated this way:


(3) $${\text{BM}}_{i}=\alpha_{i}\cdot {\text{DBH}}^{\beta_{i}}\cdot H^{\gamma_{i}}$$

where the parameters *α_i_*, *β_i_*, and *γ_i_* for BM_t_ were 0.0885, 0.625, and 1.938; likewise, for BM_b_, they were 0.0049, 0.252, and 2.736; for BM_r_, they were 0.1843, 0.758, and 1.708; and for BM_l_, they were 0.0358, 0.229, and 1.881, respectively (Lan et al., 2020). Arbor biomass carbon density (CD, t C hm^−2^) at the quadrat level was calculated using the following equation:


(4) $$\text{CD}=\sum_{j=1}^{\text{PD}/25}\sum_{i=1}^{3}\left(C_{i}\cdot {\text{BM}}_{i,j}\right)/40$$

where *j* was the *j*th tree in the quadrat; the parameter *C_i_* was the carbon coefficient for the trunk, branch, root, or leaf part of the tree, respectively, equal to 0.4817, 0.4924, 0.5046, and 0.4899 (Xu et al., 2016).

The mean and standard deviation of DBH, HWR, LAI, PD, and CD at the three elevation sites are presented in **Table 1**.

#### 2.2.2 Observed crown rainfall allocation and throughfall, stemflow, and interception ratio calculations

Four individual trees were randomly selected in each quadrat, and their stemflow was collected immediately after a rainfall event. To do this, the lower part of the trunk was simply cleared. The half-cut polyethylene rubber pipe was twined around the bark (at least two to three circles) and fixed with iron nails, and the gap between the pipe wall and trunk bark was filled with resin glue. The lower end of the water outlet was connected to a fixed small-mouth bucket used to collect the stemflow. Meanwhile, under the crown of each selected tree, four self-made simple rain-measuring barrels were randomly arranged to collect the throughfall water. Correspondingly, four self-made simple rain buckets were also positioned outside the stand to collect rainfall outside the forest. After a rainfall event, the volume (ml) of water in each water storage bucket or rain-measuring bucket was immediately collected, measured, and recorded, and then the throughfall (TF, mm), stemflow (SF, mm), and outside rainfall (*I*, mm) were calculated and converted accordingly. On the basis of a presumed water balance, crown interception (IC, mm) was calculated:


(5) IC=I−TF−SF

The throughfall, stemflow, and interception ratio (TFR, SFR, and ICR, %) were calculated as follows:


(6a) $$\text{TFR}=\frac{\text{TF}}{I}\;\times \;100\%$$

(6b) $$\text{SFR}=\frac{\text{SF}}{I}\;\times \;100\%$$

(6c) $$\text{ICR}=\frac{\text{IC}}{I}\;\times \;100\%$$

### 2.3 Data processing and statistics

#### 2.3.1 Fitting the changes in crown throughfall, trunk stemflow, and crown interception ratio in relation to outside rainfall

For the different elevation sites, on the rainfall event scale, the relationships between TFR and *I* were fitted by applying this equation:


(7a); $$\text{TFR}=a_{1}\cdot I^{a_{2}}$$

hence,


(7b) $$\text{TF}=a_{1}I^{a_{2}+1}/100$$

for which the parameters of *a*_1_ and *a*_2_ with their standard errors were estimated through non-linear regression.

Trunk stemflow only emerged when *I* was sufficiently large, and so the change in SF with *I* should be fitted using a segmental approach. When *I* ≤ *b*_2_, SFR = 0 and SF = 0; otherwise,


(8a), $$\text{SFR}=b_{1}\cdot \left(I-b_{2}\right)$$

(8b) $$\text{SF}=b_{1}\cdot \left(I-b_{2}\right)/\left(100\;\times \;I\right)$$

where the parameters *b*_1_ and *b*_2_ with their standard errors were estimated through piecewise linear regression. Thereinto, *b*_2_ was the threshold *I* when trunk stemflow emerged. The relationship between ICR and *I* could be described this way:


(9a) $$\text{ICR}=d_{2}\left(1-\text{exp}\left(-d_{1}\cdot I\right)\right)/I$$

and thus, IC is calculated as


(9b), $$\text{IC}=d_{2}\left(1-\text{exp}\left(-d_{1}\cdot I\right)\right)/100$$

where the parameters *d*_1_ and *d*_2_ with their standard errors were estimated through non-linear regression. Thereinto, *b*_2_/100 was the saturation value of IC.

#### 2.3.2 Fitting the relationship between crown rainfall allocation and forest stand variables

At the quadrat level, DBH, HWR, LAI, and PD were set as independent variables (*x*), while TFR, SFR, and ICR were designated the dependent variables (*y*). Linear regression was used to quantify the relationship between the independent and dependent variables,


(10) $$y=\alpha_{1}x+\alpha_{0}$$

The orientation and trend of the dependent variable as a function of an independent variable were judged and analyzed *via* parameter *α*_1_. Pearson’s *r* correlation coefficient and its *p*-value were also used to judge the significance.

#### 2.3.3 Fitting the relationship between crown interception ratio and arbor biomass carbon density

According to this study’s field observations, SF could be ignored because trunk stemflow contributed a small proportion to the precipitation allocation, and thus there was a complementary relationship between TFR and ICR (TFR + ICR ≈ 1). At the quadrat level, the Michaelis–Menten equation was used to describe the quantitative relationship between ICR (*y*) and CD (*x*)


(12) $$y=\varphi_{1}x/\left(\varphi_{2}+x\right)$$

Through the double-reciprocal transformation, the Lineweaver–Burk equation was obtained:


(13) $$\frac{1}{y}=\frac{\varphi_{2}}{\varphi_{1}x}+\frac{1}{\varphi_{1}}$$

Following the Lineweaver–Burk equation, after the double-reciprocal conversion of ICR (*y*) and CD (*x*), linear regression was applied. For this, the reciprocal of the intercept was the parameter *φ*_1_, whereas the slope was the ratio between the parameter *φ*_2_ and *φ*_1_.

#### 2.3.4 Numerical calculation and statistics

In this study, the functions “fitlm” and “fitnlm” in MATLAB R2017b were used to implement the linear and non-linear regressions. The non-linear fitting was judged by the coefficient of determination (*R* ^2^) and RMSE. The closer to 1 the *R* ^2^ is, and the closer to 0 the RMSE is, the better the fit of the non-linear regression model. For linear regressions, the *R* ^2^ along with the *F*-test and its *p*-value were used to test the fitness of each model. If *p<* 0.05, the fitted regression was significant; otherwise, it was deemed insignificant.

## 3 Results

### 3.1 Relationship between crown throughfall and outside rainfall at different elevation sites

**Figure 1:**
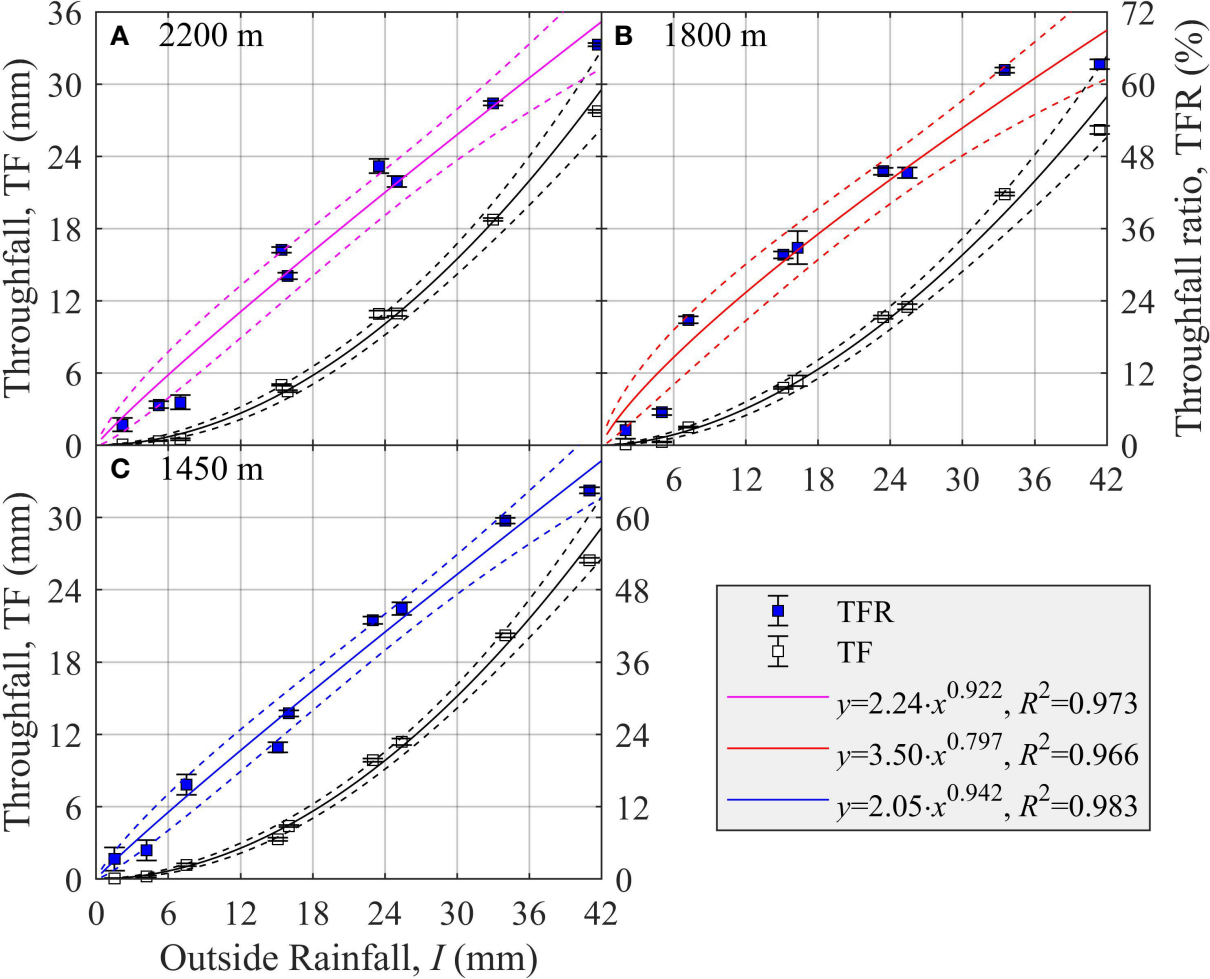
The changes in crown throughfall (TF, mm) and throughfall ratio (TFR, %) of *Picea schrenkiana* as a function of outside rainfall (*I*, mm) at three elevation sites at the northern slope of Mount Bogda, Tianshan Range, Xinjiang, China (*N* = 9). Error bars indicate the standard error of the mean (*N* = 9). Dashed lines are the upper and lower 95% predicted intervals.

Both TF and TFR increased with *I* at all three elevations of 2,200 m, 1,800 m, and 1,450 m on the northern slope of Mt. Bogda (**Figure 1**). For each, the relationship between TFR and *I* fitted using Eq. (7a) was significant: 2,200 m (*R* ^2^ = 0.973, *p<* 0.001), 1,800 m (*R* ^2^ = 0.966, *p<* 0.001), and 1,450 m (*R* ^2^ = 0.983, *p<* 0.001) (**Figure 1**). The estimated parameter *a*_1_ ( ± SE) was 2.24 ± 0.65, 3.50 ± 0.97, and 2.05 ± 0.49, while the parameter *a*_2_ ( ± SE) was 0.922 ± 0.085, 0.797 ± 0.082, and 0.942 ± 0.071 at the 2,200-m, 1,800-m, and 1,450-m elevation sites, respectively (**Figure 1**).

Accordingly, when *I* was 5 mm, 10 mm, and 25 mm, TFR ( ± SE) was 9.9 ± 3.6%, 18.7 ± 4.3%, and 43.6 ± 3.8% at 2,200 m; 12.6 ± 4.4%, 22.0 ± 4.8%, and 45.6 ± 4.0% at 1,800 m; and 9.4 ± 2.8%, 18.0 ± 3.4%, and 42.6 ± 3.0% at 1,450 m, respectively (**Figure 1**). Thus, for a given *I*, TFR was largest and smallest at 1,800 m and 1,450 m, respectively (**Figure 1**).

### 3.2 Relationship between stemflow and outside rainfall at different elevation sites

**Figure 2:**
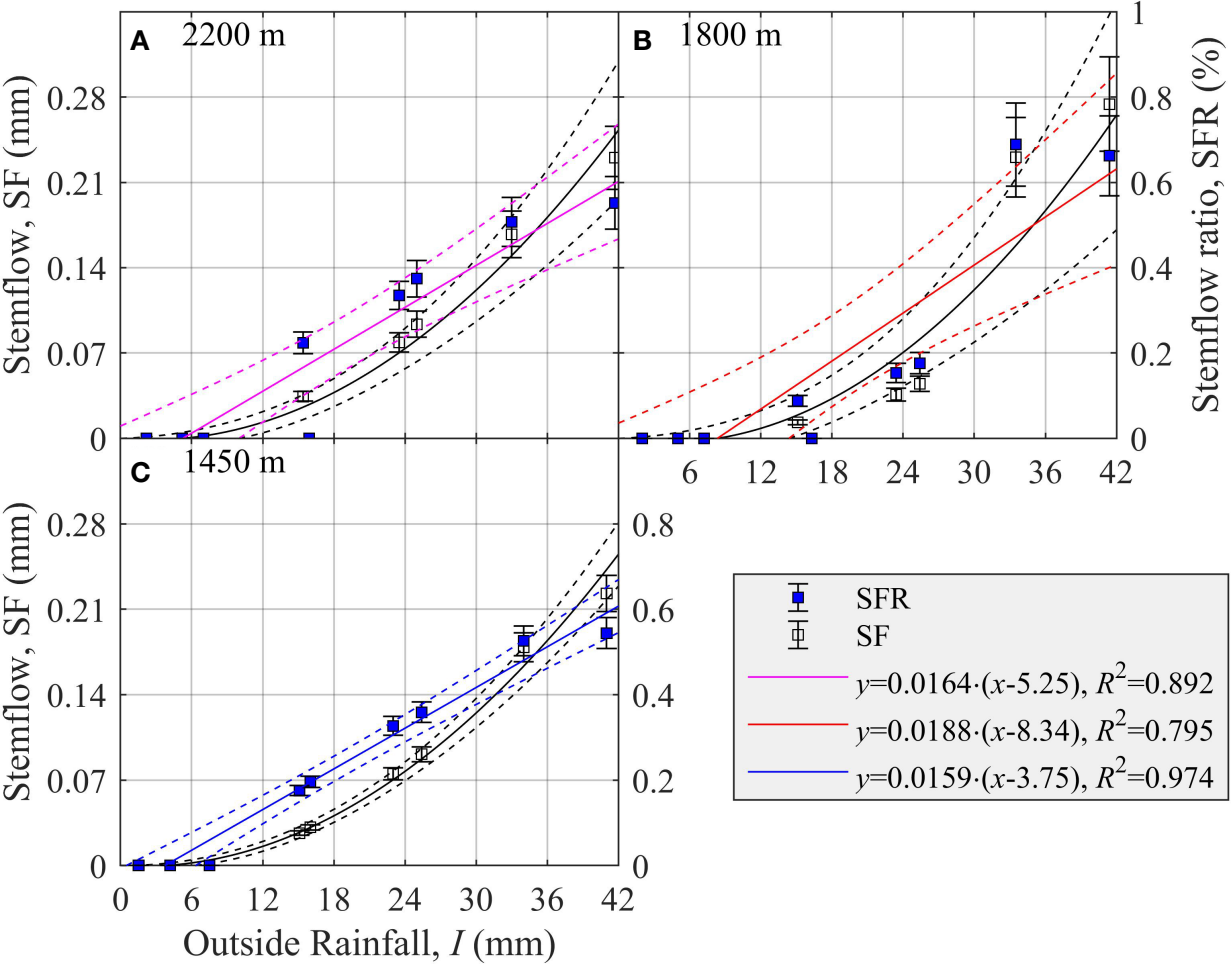
Changes in stemflow (SF, mm) and stemflow ratio (SFR, %) of *Picea schrenkiana* as a function of outside rainfall (*I*, mm) at three elevation sites on the northern slope of Mount Bogda, Tianshan Range, Xinjiang, China. Error bars indicate the standard error of the mean (*N* = 9). Dashed lines are the upper and lower 95% predicted intervals.

Both SF and SFR increased significantly with *I* at all three elevation sites (**Figure 2**). The relationship between SFR and *I* fitted *via* Eq. (8a) was found significant at 2,200 m (*R* ^2^ = 0.892, *p<* 0.001), 1,800 m (*R* ^2^ = 0.795, *p<* 0.001), and 1,450 m (*R* ^2^ = 0.974, *p<* 0.001) (**Figure 2**). The estimated parameter *b*_1_ ( ± SE) was 0.0164 ± 0.0022, 0.0188 ± 0.0036, and 0.0159 ± 0.0010, while the parameter *b*_2_ ( ± SE) was 5.25 ± 2.42 mm, 8.34 ± 3.13 mm, and 3.75 ± 1.22 mm at the three elevation sites of 2,200 m, 1,800 m, and 1,450 m, respectively (**Figure 2**). Thus, stemflow first emerged at the 1,450-m elevation site, and then at the 2,200-m elevation site, and last at the 1,800-m elevation site (**Figure 2**). However, according to the parameter *b*_1_, SFR accelerated the most at 1,800 m elevation, followed by the 2,200-m elevation, and least at the 1,450-m elevation (**Figure 2**).

When *I* was 42 mm, SFR was 0.60 ± 0.13%, 0.63 ± 0.22%, and 0.61 ± 0.06% at the three elevation sites of 2,200 m, 1,800 m, and 1,450 m, respectively (**Figure 2**). Thus, they are all ca. 0.60%, and so *I* is in the range of 0–42 mm, and SFR was determined by the threshold *I* of the parameter *b*_2_. That is, the larger threshold *I*, the smaller SFR was when *I* was the same (**Figure 2**).

### 3.3 Relationship between crown interception and outside rainfall in different elevation sites

**Figure 3:**
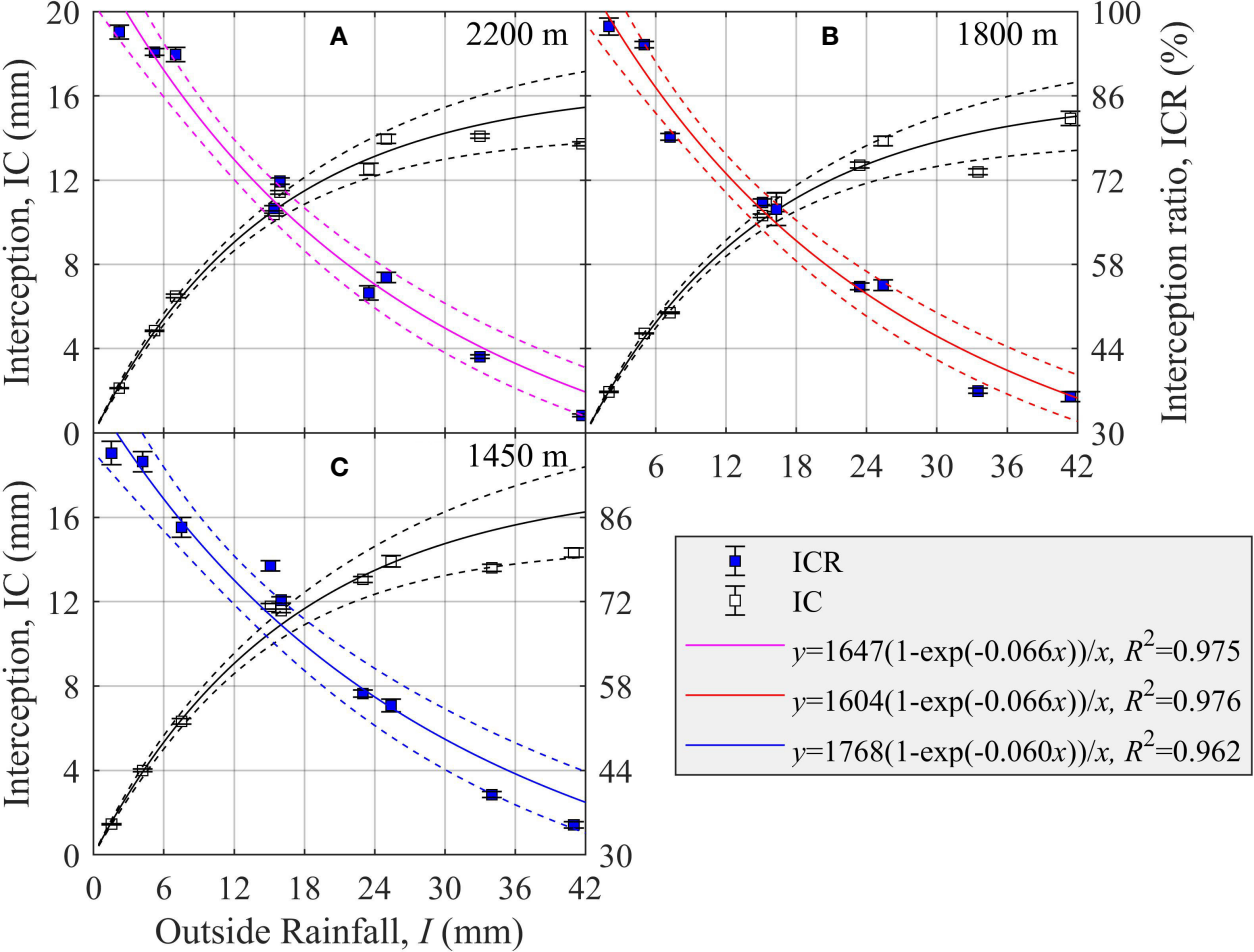
Changes in the crown interception (IC, mm) and interception ratio (ICR, %) of *Picea schrenkiana* as a function of outside rainfall (*I*, mm) at three sites on the northern slope of Mount Bogda, Tianshan Range, Xinjiang, China. Error bars indicate the standard error of the mean (*N* = 9). Dashed lines are the upper and lower 95% predicted intervals.

As ICR decreased, the IC increased but its acceleration decreased gradually with *I* at the three elevations (**Figure 3**). Thus, there was an asymptote for IC when *I* was large enough and its acceleration was close to 0. There was a significant relationship between ICR and *I* as fitted by Eq. (9a) at each elevation site: 2,200 m (*R* ^2^ = 0.975, *p<* 0.001), 1,800 m (*R* ^2^ = 0.976, *p<* 0.001), and 1,450 m (*R* ^2^ = 0.962, *p<* 0.001) (**Figure 3**). The estimated parameter *d*_1_ ( ± SE) was 0.066 ± 0.005, 0.066 ± 0.005, and 0.060 ± 0.006, whereas the parameter *d*_2_ ( ± SE) was 1,647 ± 100, 1,604 ± 96, and 1,768 ± 136 at the three elevation sites of 2,200 m, 1,800 m, and 1,450 m (**Figure 3**). Hence, *d*_2_/100 represents the saturation value of IC, which was 16.47 ± 1.00 mm, 16.04 ± 0.96 mm, and 17.68 ± 1.36 mm at the 2,200-m, 1,800-m, and 1,450-m elevation sites, respectively (**Figure 3**).

Accordingly, when *I* was 5 mm, 10 mm, and 25 mm, ICR ( ± SE) was 93.1 ± 4.9%, 80.0 ± 3.5%, and 53.4 ± 4.0% at 2,200 m; 90.1 ± 4.6%, 77.4 ± 3.3%, and 51.8 ± 3.8% at 1,800 m; and 91.6 ± 5.7%, 79.7 ± 4.2%, and 54.9 ± 4.8% at 1,450 m, respectively (**Figure 3**). Thus, ICR was smallest at the 1,800-m elevation site, and when *I* was low, ICR was largest at the 2,300-m elevation site, and when *I* was heavy, ICR was largest at the 1,450-m elevation site (**Figure 3**).

### 3.4 Relationships between TFR, SFR, ICR, and forest stand factors DBH, HWR, PD, and LAI

**Figure 4:**
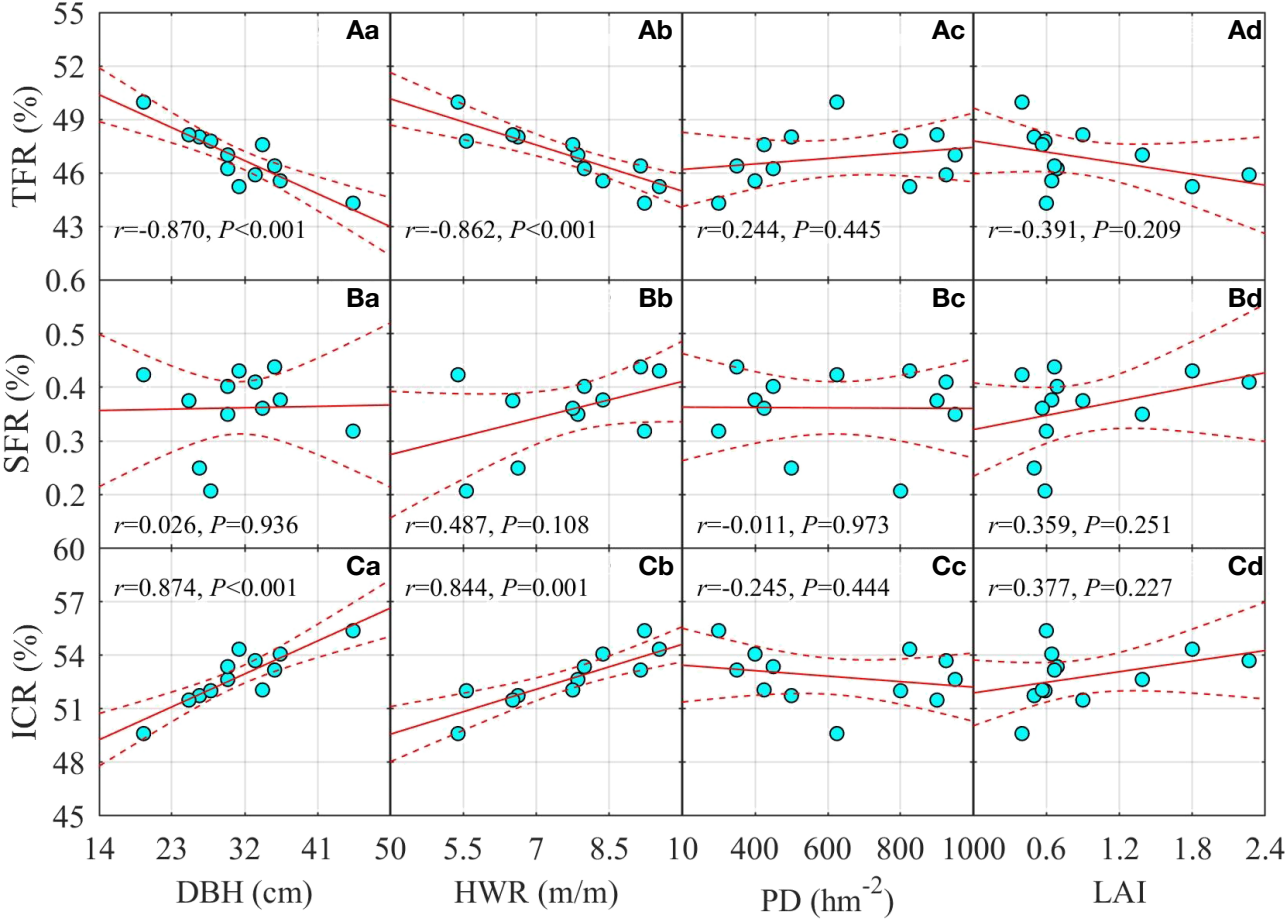
Changes in the crown throughfall, stemflow, and interception ratio (TFR, SFR, and ICR) of *Picea schrenkiana* with DBH, crown height-to-width ratio (HWR), population density (PD, hm^−2^), and leaf area index (LAI) on the northern slope of Mount Bogda, Tianshan Range, Xinjiang, China (*N* = 12). Dashed lines are the upper and lower 95% predicted intervals.

TFR decreased significantly with DBH (*r* = –0.870, *p<* 0.001) and HWR (*r* = –0.862, *p<* 0.001), yet increased insignificantly with PD (*r* = 0.244, *p* = 0.445), but decreased insignificantly with LAI (*r* = –0.391, *p* = 0.209, **Figure 4Aa–d**). By contrast, ICR increased significantly with both DBH (*r* = 0.874, *p<* 0.001) and HWR (*r* = 0.844, *p<* 0.001), and decreased insignificantly with PD (*r* = –0.245, *p* = 0.444) and increased insignificantly with LAI (*r* = 0.377, *p* = 0.227, **Figure 4C**a–d). The trends of SFR were the same as those of ICR with respect to DBH, HWR, PD, and LAI, but the correlations were considerably weaker. Finally, ICR increased insignificantly with DBH (*r* = 0.026, *p* = 0.936), HWR (*r* = 0.487, *p* = 0.108), and LAI (*r* = 0.359, *p* = 0.251), but decreased insignificantly with PD (*r* = –0.011, *p* = 0.973, **Figure 4Ba–d**).

### 3.5 Relationship between ICR and CD

**Figure 5:**
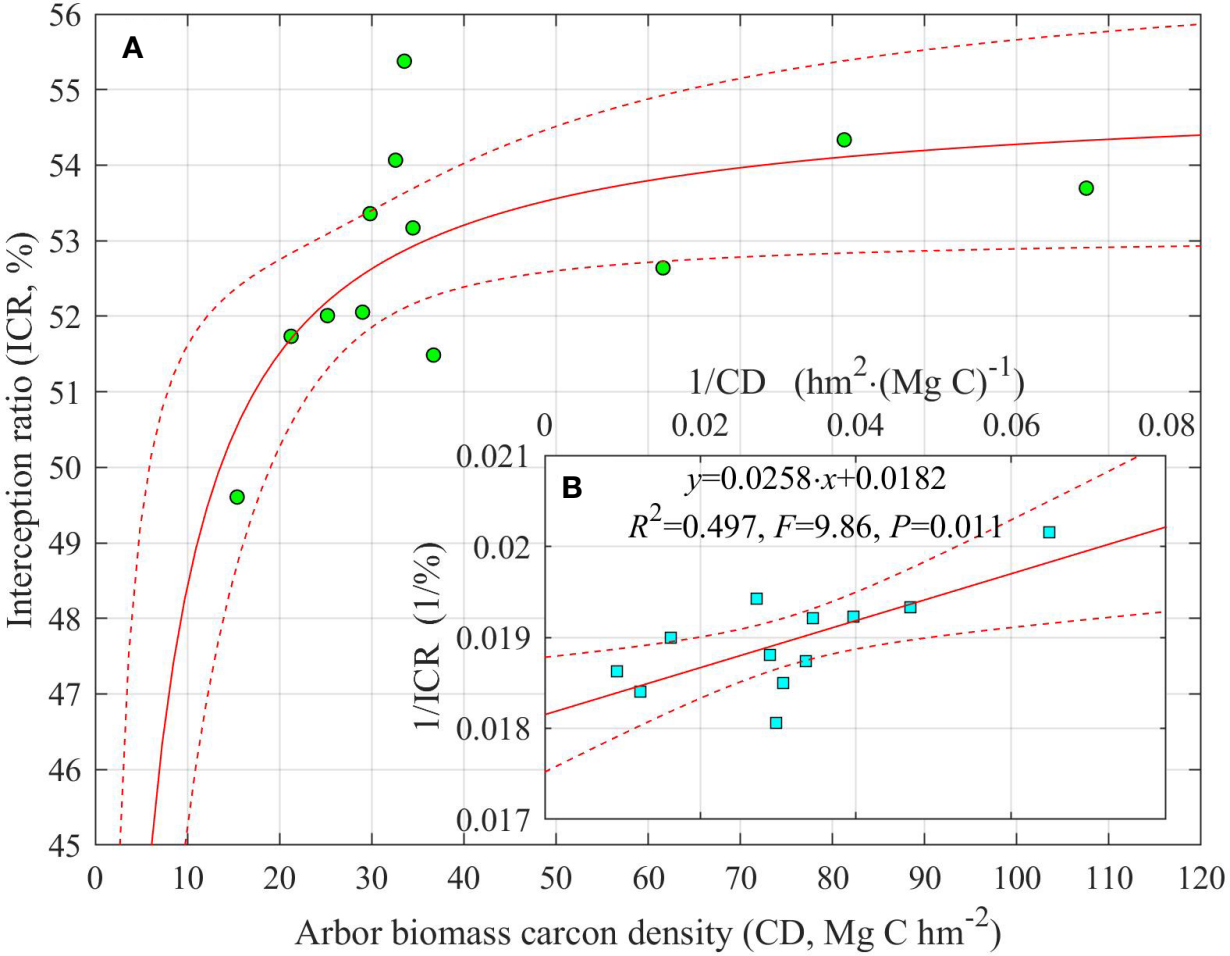
**(A)** Change in the crown interception ratio (ICR, %) of *Picea schrenkiana* with arbor biomass carbon density (CD, t C hm^−2^) on the northern slope of Mount Bogda, Tianshan Range, Xinjiang, China (*N* = 12). **(B)** The double-reciprocal linear relationship between CD and ICR. Dashed lines are the upper and lower 95% predicted intervals.

With increasing CD, ICR increased sharply at first and then gradually tapered off (**Figure 5A**). After the double-reciprocal transformation, there was a significant linear relationship (*R* ^2^ = 0.497, *F* = 9.86, *p* = 0.011), whose slope and intercept ( ± SE) were 0.0258 ± 0.0082 and 0.0182 ± 0.0003, respectively (**Figure 5B**). Thus, the maximum predicted ICR was 55.01 ± 0.86% (**Figure 5A**). When CD was 1.36 ± 0.49 Mg C hm^−2^, ICR was the half of its maximum value; when CD was 20 Mg C hm^−2^, ICR was 51.51 ± 1.24% or 93.6 ± 2.3% of the maximum ICR attainable (**Figure 5A**).

## 4 Discussion

### 4.1 Forest stand factors influencing crown rainfall allocation

Tree crown allocation of rainfall is a crucial yet an understudied component of the forest water balance (Allen et al., 2020). The complex crown structure determines the allocation pattern of water and its relative quantities, which can change depending on the age and species of trees, and the biome, and other factors (Gonzalez-Ollauri et al., 2020). For example, the maximum interception and held rainfall of broad-leaf, scale-leaf, and needle-leaf types is about 2.0 mm, 3.7 mm, and 4.3 mm, respectively, differences that translate into a significantly higher rainfall interception effect from the crowns of coniferous than broad-leaf trees; hence, the crown throughfall ability in coniferous trees is weaker than that in broad-leaved trees (Klamerus-Iwan et al., 2020). During the observation period of the present study, TFR ranged from 44.3% to 50.0%, while ICR ranged from 49.6% to 55.4% for a pure coniferous forest. Also, due to the large interception and attachment effect, the SFR did not exceed 0.5% (**Figure 4**).

The rainfall interception effect of a forest stand is mainly affected by crown interception, retention, and absorption capacity; this is manifested as the interception of rainfall by leaves and branches, retention in branches/buds/leaf axils, and water absorption by relative dry branch/leaf surfaces. In general, at the individual tree level, the greater the surface area, the coarser and drier the surface of the above-ground protrusions (branches and leaves) per unit area of crown foliage and the stronger the crown capture, retention, and absorption capacity tend to be. Accordingly, in this study, although insignificant, ICR increased but TFR decreased with LAI (**Figure 4**), which is the amount of leaf surface area per unit land surface. Both ICR and TFR were more significantly correlated with DBH, a measure of tree size and age (**Figure 4**). In the process of forest stand development, with the thickening of the trunk DBH, the competition between adjacent individuals intensified, influencing to a greater extent the growth in crown width than height of trees. Therefore, HWR increased gradually, and there was a positive correlation between HWR and DBH (*N* = 12, *r* = 0.742, *p* = 0.006). Consequently, HWR was significantly positively correlated with ICR but significantly negatively correlated with TFR (**Figure 4**).

In addition, we did not find a significant positive correlation between LAI and either SFR or HWR (**Figure 4Bb**), a result that does not support the commonly held notion that horizontal crown expansion increases the rainfall harvesting capacity of crowns (André et al., 2008; Hofhansl et al., 2012). However, in the Cerrado savanna of Brazil, South America, regardless of individual density, the interaction between HWR and DBH in three groups of trees differing in bark smoothness compensated for the adverse effects of bark roughness on stemflow (Tonello et al., 2021). It is generally considered that HWR is conducive to the formation and amplification of stemflow and that it could also reflect the branching angle of trees; however, measuring branching angles is very difficult to do in the field (Sadeghi et al., 2020). Along with the development of a forest stand, irrespective of whether or not the branching angle of its trees changed due to the competition between adjacent individuals, the relative growth rate of tree height must surpass that of crown width. Therefore, HWR gradually magnified, PD declined, and LAI also rose, which inevitably resulted in the higher SFR and ICR but lower FTR in the studied stands (**Figure 4**).

### 4.2 Effect of outside rainfall on crown rainfall allocation

Throughfall could form through the gaps between pine needles of a tree without being intercepted by the crown. However, there is an evident temporal lag effect in the formation of stemflow, because it only occurs after rainfall has been intercepted by pine needles and branches above a certain crown threshold. Therefore, in addition to biological factors, outside rainfall is the most important abiotic environmental factor affecting the forest crown allocation of rainfall (Zimmermann et al., 2015). As the amount of outside rainfall increased, the proportion of rainfall allocated among throughfall, stemflow, and interception routes varied. The basic rule discerned for *P. schrenkiana* stands was as TFR and SFR increase, the ICR decreases. However, there is a certain maximum for IC (**Figures 1**–**3**).

Outside rainfall is recognized as the driving force for changes to IC, such that IC increases at first and then tends to stabilize (Macinnis-Ng et al., 2014; Wan et al., 2016; Li et al., 2020). The process of ICR’s change in response to *I* could be divided into three stages (Dang et al., 2005): rapid increase (*I<* 5* mm*, ICR ≥ 36.1%), gradual increase (5 ≤ *I<* 25* mm*, 20.4% ≤ ICR< 36.1%), and stable (*I* ≥ 25 mm, ICR< 20.4%). Analogously, for the three levels of outside rainfall of 0–5, 5–25, and 25–40 mm, the corresponding relative change in IC was 0.90, 0.42, and 0.13 mm/mm (**Figure 3A**). Notably, stemflow did not occur when little rain fell (i.e., 0–5 mm) (**Figure 2A**), and thus almost all the rainfall intercepted by the tree crown was converted into IC, keeping ICR above 93.1 ± 4.9% (**Figure 3A**). Finally, IC did not increase though ICR gradually decreased with *I*. As a result, IC at the 2,300-m, 1,800-m, and 1,450-m elevation sites remained at 16.47 ± 1.00 mm, 16.04 ± 0.96 mm, and 17.68 ± 1.36 mm, respectively (**Figure 3**). There was a linear relationship between IC and *I* (Zhang et al., 2015a), which could be related to the fact that the observed *I* was not large enough to reach a stable stage.

All biotic and abiotic factors that could variously affect IC should influence the generation and amplification of SF, but there are also differences in this respect. For example, a small branching angle is conducive to the generation of SF but not conducive to IC, while bark thickness and roughness are conducive to IC but not conducive to SF (Hu et al., 2018). This may be related to forest crown allocation of rainfall, which may be divided into two steps: first, rainfall interception occurred, and then once the interception capacity was exceeded, a portion of it was collected onto the trunk to form SF, while another portion dropped directly from the crown to form TF (Gonzalez-Ollauri et al., 2020). Therefore, in the process of influx to the trunk, the dryness and hygroscopicity of the crown and bark as well as the roughness of the bark and the branching angle could have jointly affected stemflow, eventually leading to the existence threshold of SF (Macinnis-Ng et al., 2014). For example, in this study, at the 2,200-m, 1,800-m, and 1,450-m elevations, when SF emerged, outside rainfall was 5.25 ± 2.42 mm, 8.34 ± 3.13 mm, and 3.75 ± 1.22 mm, respectively (**Figure 2**).

### 4.3 Differences in crown rainfall allocation among the three elevation sites

In this study, across the 2,200-m, 1,800-m, and 1,450-m elevation sites, their forest crown allocation of rainfall clearly differed, mainly for the threshold value of *I* when SF emerged and the saturation of IC. These disparities should be explainable in terms of local stand factors at the three elevations. For the same amount of *I*, ICR was highest at 1,800 m, followed by 2,300 m, and the lowest was at 1,450 m (**Figure 1**), a ranking related to different average values of DBH, HWR, and LAI among three sites (**Table 1**) as implied by the positive correlation of ICR with DBH, HWR, and LAI (**Figure 4**).

At all three elevation sites, the threshold value of *I* when stemflow emerged showed a trend opposite to the acceleration of SF with *I* (**Figure 2**). That is, the rank ordering among sites for their threshold of outside rainfall when stemflow emerged was 1,450 m > 2,200 m > 1,800 m, but vice versa (1,800 m > 2,200 m > 1,450 m) for the acceleration of SF (**Figure 2**), which may be related to the stand differences in DBH, HWR, and LAI across elevation (**Table 1**). There was no significant negative correlation between SFR and LAI (**Figure 4**), likely because just two stages of forest crown allocation of rainfall were involved: (1) when the interception and capture ability was within the water-holding ability, and (2) when the interception and capture ability exceeded that holding ability. Due to the greater DBH, growth in tree height, and enhanced bark water absorption, the first stage above, the more important one, could offset the adverse effects of the latter stage on stemflow, leaving SFR only very weakly positively correlated with DBH and HWR (**Figure 4**).

The saturated values of IC at the 2,200-m, 1,800-m and 1,450-m elevation sites were 16.47 ± 1.00 mm, 16.04 ± 0.96 mm, and 17.68 ± 1.36 mm, respectively (**Figure 3**), this order matching the DBH, HWR, and LAI (**Table 1**). ICR was positively correlated with DBH, HWR, and LAI (**Figure 4**). Yet, when *I* was small, ICR at 2,200 m surpassed that at 1,450 m (**Figure 3**), which may be related to the fact that stemflow emerged later in the former (at 5.25 ± 2.42 mm) than latter (at 3.75 ± 1.22 mm). Rainfall captured by the crown did not exceed the interception capacity that fell to form throughfall or that was collected to form stemflow.

### 4.4 Trade-off between spline forest water and carbon ecosystem service in arid areas

Although mountains cover less than 1/5 of the earth’s land surface, they feed 50% of the world’s population and provide 80% of terrestrial fresh water resources, and even in some arid and semi-arid areas provide 90% of fresh water resources (Wang et al., 2001a; Wang et al., 2011). Especially in Xinjiang of China, water shortage has become a bottleneck problem now restricting local economic and social development (Sun et al., 2021). As an important water conservation forest in Xinjiang, Tianshan Mountain forest has various pressing problems, such as its single tree species dominance, stand structure, and difficult renewal; thus, soil erosion and water resource shortages are accompanied by a vicious cycle of ecological environment degradation that worsens over time. Thus, how to achieve greater runoff production and improved water and soil conservation functions through reasonable ecological protection and restoration has become a major problem that needs to be solved now (Aru et al., 2019). However, from a data analysis of 504 typical watersheds and more than 600 observation points worldwide, combined with the simulation results of ecological and economic models, it was concluded that enlargement of the carbon sink by afforestation can substantially reduce the production of runoff, being reduced by as much as 52% (Wang et al., 2011). In the forest and grassland zone at middle elevation in the Tianshan Mountains, the runoff yield varies greatly among different types of vegetation cover, as follows in descending order: bare land (32.74 m^3^·hm^−2^), grassland (17.15 m^3^·hm^−2^), natural forest (9.78–17.70 m^3^·hm^−2^), and artificial forest (10.26–11.18 m^3^·hm^−2^). With an increase in age of forest stands and their crown density, the runoff yield decreases gradually, amounting to only 54.1%–29.9% of bare land. Yet, the runoff yield of artificial forest was only equivalent to the lower limit of natural forest (Sun et al., 2021).

In this study, with the increase of CD, at first ICR increased sharply and then its acceleration declined and eventually did not increase any more (**Figure 5**). This result indicates that water conservation capacity and biomass carbon fixation exhibit a synergistic increasing trend when the forest stand is young. However, when the stand age exceeded a certain threshold, ICR did not increase with the accumulation of CD (**Figure 5**). Thus, at least from the perspective of rainfall allocation by the crown, when the stand age of *P. schrenkiana* reaches a certain stage, its carbon sequestration did not influence the complementary relationship between water conservation (IC) and runoff production (TF) in the forest. The accumulation of biomass carbon in forest ecosystems is mainly embodied in the expanding tree timber volume (coarse roots, trunks, and branches) whereas functional organs such as fine roots and leaves do not always increase, having asymptotic values, rendering their biomass rather small relative to the total individual tree biomass (Thomas, 1996). Fine roots absorb water and nutrients, whereas leaves assimilate CO_2_ through photosynthesis and discharge H_2_O through transpiration. When forest stand was at a mature stage and resources are fully utilized, the quantity and biomass of those functional organs appeared quasi-static because a dynamic equilibrium between their growth and death is maintained. Thus, despite the trees’ accumulation of biomass carbon, the runoff yield as well as water consumption of the stand remained relatively stable. However, artificial afforestation to expand the area of woodland will lessen the area of other vegetation types, such as grassland and shrubland. While this land use change would reduce runoff yield, it would increase water consumption in the upstream runoff-producing mountains and concomitantly reduce the amount of water available to reach the downstream oasis of this inner arid zone of Eurasia.

## Data availability statement

The original contributions presented in the study are included in the article/supplementary material. Further inquiries can be directed to the corresponding author.

## Author contributions

All the authors contributed to the study conceptualization and performed the experiments. YW and X-JZ conceived the study. SZ and X-JZ conducted the experiments, analyzed the data, and drafted the manuscript. LY critically reviewed and edited the manuscript. All authors contributed to the article and approved the submitted version.
